# Cortical tissue loss and major structural reorganization as result of distal middle cerebral artery occlusion in the chronic phase of nude mice

**DOI:** 10.1038/s41598-019-43341-0

**Published:** 2019-05-02

**Authors:** Anuka Minassian, Marina Dobrivojevic Radmilovic, Stefanie Vogel, Michael Diedenhofen, Melanie Nelles, Maren Stoeber, Dirk Wiedermann, Mathias Hoehn

**Affiliations:** 1 In-vivo-NMR Laboratory, Max Planck Institute for Metabolism Research, Cologne, Germany; 2 Department Radiology, Leiden University Medical Center, Leiden, Netherlands

**Keywords:** Stroke, Neurodegeneration

## Abstract

The stroke model of distal middle cerebral artery occlusion is considered a reliable stroke model with high reproducibility and low mortality rate. Thus, it is preferred for assessments of therapeutic strategies, in particular for neurorepair and regeneration studies. However, present literature has reported only on the lesion behavior and behavioral deficits during the acute and subacute phase of maximally three weeks. We have here aimed to characterize the lesion expansion and consequent, potential tissue displacements using structural magnetic resonance imaging modalities, histology, and behavioral tests, during the chronic time window of 12 weeks following stroke induction. We found a severe cortical thinning resulting in 15% tissue loss of the ipsilateral cortex by 6 weeks. After two weeks, massive hippocampus displacement was found, into the cortical tissue void and, in this process, pushing the corpus callosum to the brain surface showing an almost radial direction towards the surface. These massive chronic morphological changes and rearrangements, not known from other stroke models, have relevant consequences for decision of stem cell graft placement for cerebral regeneration to assure persistent graft vitality during a longitudinal investigation in the chronic phase.

## Introduction

Stroke remains the major cause for permanent disabilities and ranks among the most common causes of death within the Western countries. For the characterization of the pathophysiological changes and the assessment of new therapeutic strategies, various stroke rodent models have been developed. Among those, intraluminal filament occlusion of the middle cerebral artery (MCAO) is the most widely used model. However, for the investigation of stem cell mediated regeneration, the transient occlusion of MCA (tMCAO) produces lesion sizes that challenge the long-term survival of grafted stem cells^1^. An alternative model with smaller lesion volume, limited to the cerebral cortex is the distal occlusion of the MCA (dMCAO) by cauterization. This model has been shown to be highly reproducible and robust, with low mortality^2,3^, an important prerequisite to assess neurorepair and stem cell-mediated regeneration processes. Such processes are slow and require long-term observation during the chronic phase of the lesion development, but to date the dMCAO model has been characterized only during the acute and subacute phase, during the first three weeks only^4–7^ or even less^2,3,8–10^ after stroke induction.

We, therefore, decided to carefully characterize the long-term structural changes in the distal MCA occlusion model in immune-deficient nude mice, with the intention to serve as later model for stem cell grafting in this stroke model. We aimed to assess the spontaneous dynamics of the lesion development and of morphological rearrangement processes during the chronic phase of 12 weeks following dMCAO to provide a detailed time profile of the spontaneous situation. These observations will serve later investigations as the necessary background to determine optimal protocol conditions for therapeutic strategies. For this purpose, we have non-invasively imaged the brain changes after dMCAO during 12 weeks with high-resolution anatomical magnetic resonance imaging (MRI).

## Results

### Temporal profile of lesion development

**Figure 1:**
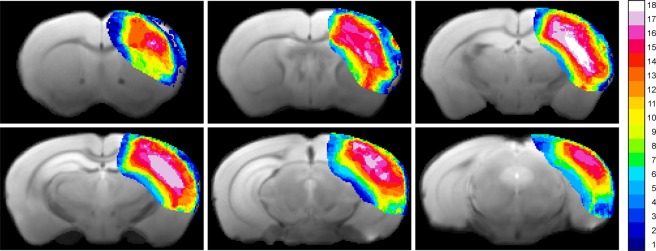
Incidence maps of ischemic lesion extent at 48 h. T2-weighted multi-slice MR imaging was performed at 48 h after dMCAO in 18 animals which were carried through the whole 12 weeks observation period. Coregistration with a mouse brain template and superposition of the hyperintense region at this time point onto the template data set indicated a stable and robust ischemic region in the cortex covering a large part of the ipsilateral cortex.

**Figure 2:**
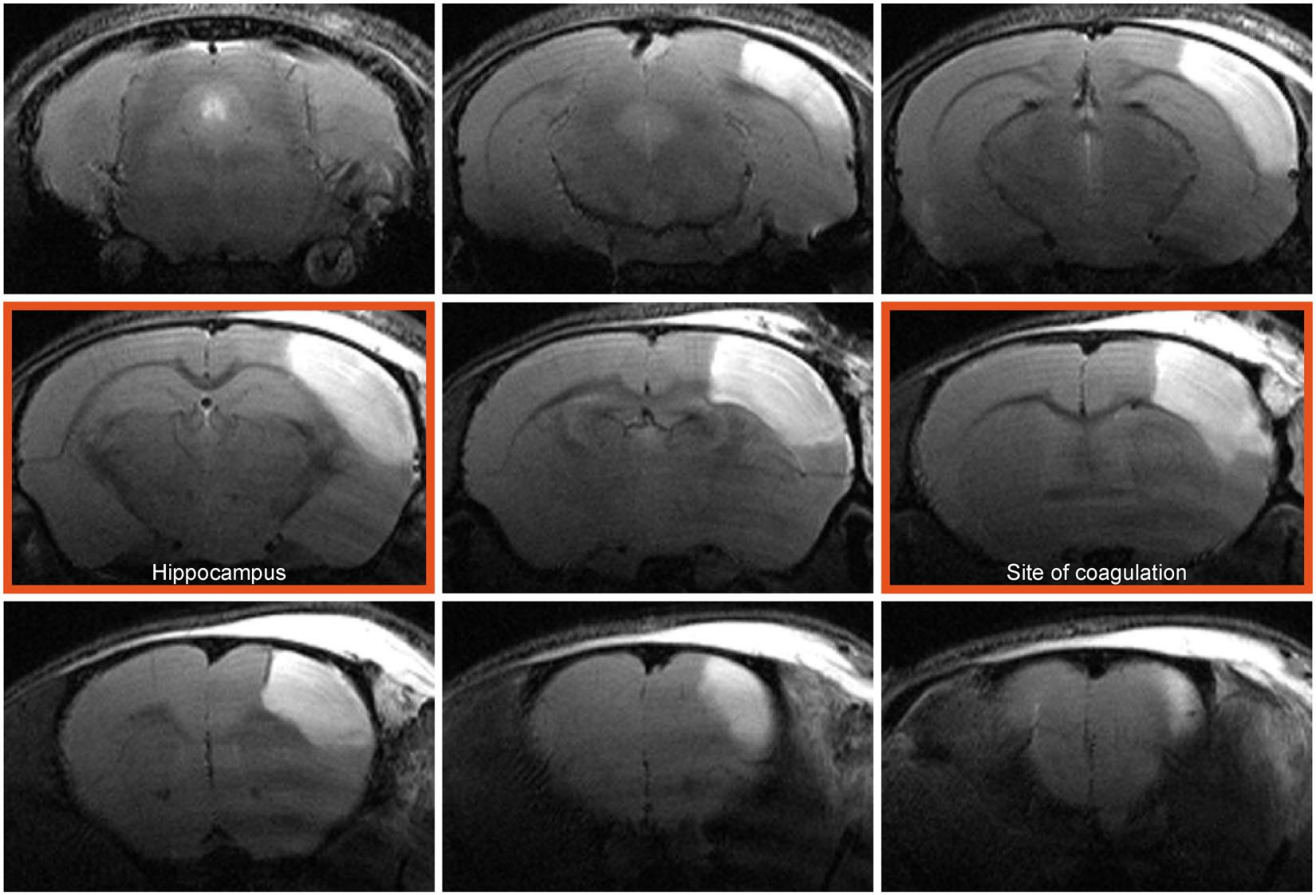
Cortical lesion depiction at the acute time point. A representative example of T2-weighted multi-slice MRI of a mouse 48 hours after dMCAO shows pronounced hyperintense regions in the right cortex covering a major part of the caudo-rostral extent. The image planes at the site of coagulation and the site where the hippocampal distortion is maximal are marked with a red frame. These two planes are used for further detailed analysis of the temporal development of the pathophysiological changes (cf. Fig. 3).

**Figure 3:**
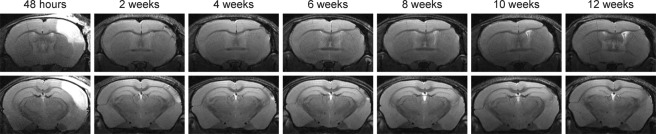
Development of the lesion during 12 weeks. T2-weighted MR images of two selected coronal positions through the brain of a representative mouse are shown at 48 h and for time points every two weeks following dMCAO. The slice positions were selected as described in Fig. 2. The hyperintensity at 48 h quickly fades away, and only in the lower row at the level of the hippocampus a small hyperintense spot at the cortical surface remains at later times. In this plane, the movement of the hippocampus towards the brain surface with time is clearly noted. In the upper row of images at the level of the MCA coagulation (cf Fig. 2), the increasing thinning of the cortex is visible together with the pronounced bending of the corpus callosum towards the brain surface on the site of coagulation.

In total, 18 mice received distal occlusion of the right middle cerebral artery. Successful occlusion was assessed at 48 h after dMCAO by T2-weighted MRI, and the extent of the ischemic territory was found to be reproducible and stable across the group of animals, as depicted in the T2 incidence maps of Fig. 1. The vasogenic edema in the affected tissue covers a large extent of the dorsolateral cortex across the rostro-caudal axis, as shown on a multi-slice set of T2-weighted images for one representative animal in Fig. 2. The affected tissue largely expands into the white matter of the corpus callosum. Two sections, at the site of the MCA coagulation and at the site of the hippocampus (Fig. 2), have been selected to demonstrate the lesion development in the chronic phase over twelve weeks (Fig. 3). A thinned cortex becomes clearly visible on the ischemic hemisphere already after 2 weeks. At week 4, parts of the cortex at the coagulation site have vanished completely so that the white matter reaches the brain surface (Fig. 3 upper row). In the posterior position at the level of the hippocampus (Fig. 3, lower row), a hippocampal swelling and movement on the ipsilateral side is visible beginning at week 2. With progressive cortical thinning during the following weeks, the hippocampus moves further into the void of the lost cortical tissue starting from week 8 on. A small hyperintense tissue area is still seen at the lateral cortical edge of the lesion at week 2; it decreases over the following weeks and has completely disappeared by week 8.

**Figure 4:**
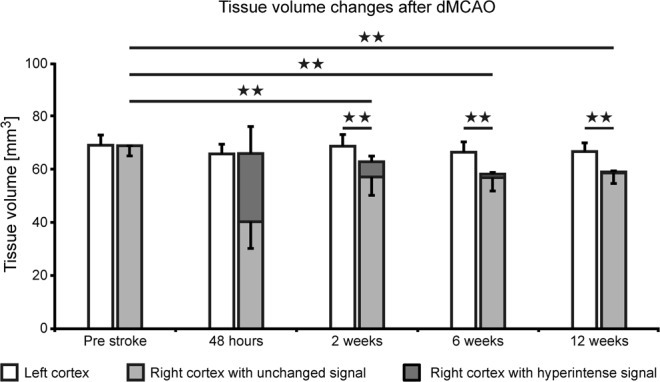
Cortical volume changes after dMCAO. Quantitative analysis of the cortical tissue volume with hyperintensity (dark grey bar) and of the normal appearing tissue (light grey bar) is presented for the whole 12 weeks observation after dMCAO. While the hyperintensity is maximal at 48 hours with 38 ± 15% of cortical volume, it quickly decreases and reaches values of 0.6 ± 0.5% at week 12. Already visible at week 2 is the loss of cortical tissue, as the sum of light and dark grey bars are significantly less than the cortical volume of the contralateral hemisphere. This tissue loss further increases with time. Error bars represent standard deviation. All individual volume data of all stroke animals at all time points are compiled in the Supplement Table 1. Error bars are presented as SD.

Thus, the lesion is characterized by two components: (i) the ischemic/necrotic cortical tissue, visible in the subacute phase as the hyper- and hypo-intense areas at week 2 and (ii) the atrophic volume of tissue loss, beginning already at week 2 but increasing continuously over time at the expense of the originally present, ischemic tissue. This substantial loss of cortical tissue explains the shift of the white matter and the hippocampus, both reaching the brain surface in this region. Quantitative analysis of this development of tissue loss is depicted in Fig. 4, clearly showing the decrease of cortical volume on the ipsilateral side with time. Also noteworthy from this quantitative analysis, the volume of hyperintensity at 48 h after stroke induction overestimates the final damage, covering a larger volume (38 ± 15% of healthy cortex) than the final tissue loss, reached at week 6 and remaining unchanged later, which amounts to 15 ± 5%.

**Figure 5:**
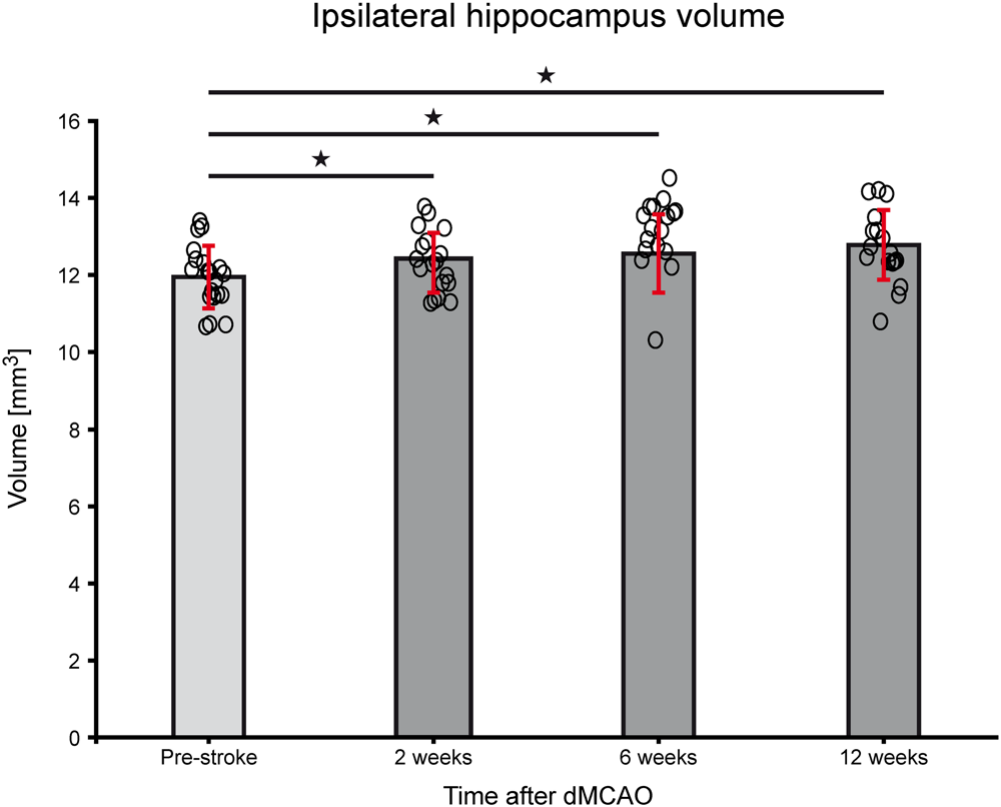
Hippocampal volume changes after dMCAO. Quantitative analysis of hippocampus volume of the ipsilateral hemisphere was performed using manual outlines and coregistration to mouse brain atlas. The volume increase of the ipsilateral hippocampus after distal MCA occlusion, visualized on the MR images (cf Fig. 3) is statistically significant for week 2 (p = 0.033), for week 6 (p = 0.018) and week 12 (p = 0.007), relative to pre-stroke. All individual volume data of all stroke animals at all time points are compiled in the Supplement Table 2. Error bars are presented as SD.

Using the manual outline of the hippocampus in all image planes and at all time points, as described in the MRI data processing section, the ipsilateral hippocampal volume was calculated over time. These results show a convincing volume increase on the ischemic hemisphere during the chronic stroke phase, as presented in Fig. 5. At the end of the 12 weeks observation period, ipsilateral hippocampal volume had swollen by 10% relative to a normal hippocampus volume pre-stroke.

In all 12 sham operated animals, no hyperintensity is observed at 48 h except for a minute spot on the cortex at the site where the forceps touch the brain surface during sham coagulation (Suppl. Fig. 1). The timeline over the full 12 weeks is shown (Suppl. Fig. 2) for the same slice positions as for the animals with distal occlusion of the MCA. At the position of the hyperintensity at 48 h, tissue loss is noted restricted to a very small region, while the position of the corpus callosum does not change at all. Also, the hippocampus does not shift towards the brain surface (Suppl. Fig. 2).

### Sensorimotor behavior tests

During the first two weeks after stroke induction by dMCAO, we used the corner test, the rotating beam test and the rotarod test to assess functional deficits caused by the stroke lesion and to unravel which of these tests is most sensitive for future longitudinal investigations of therapeutic strategies in this animal model.

**Figure 6:**
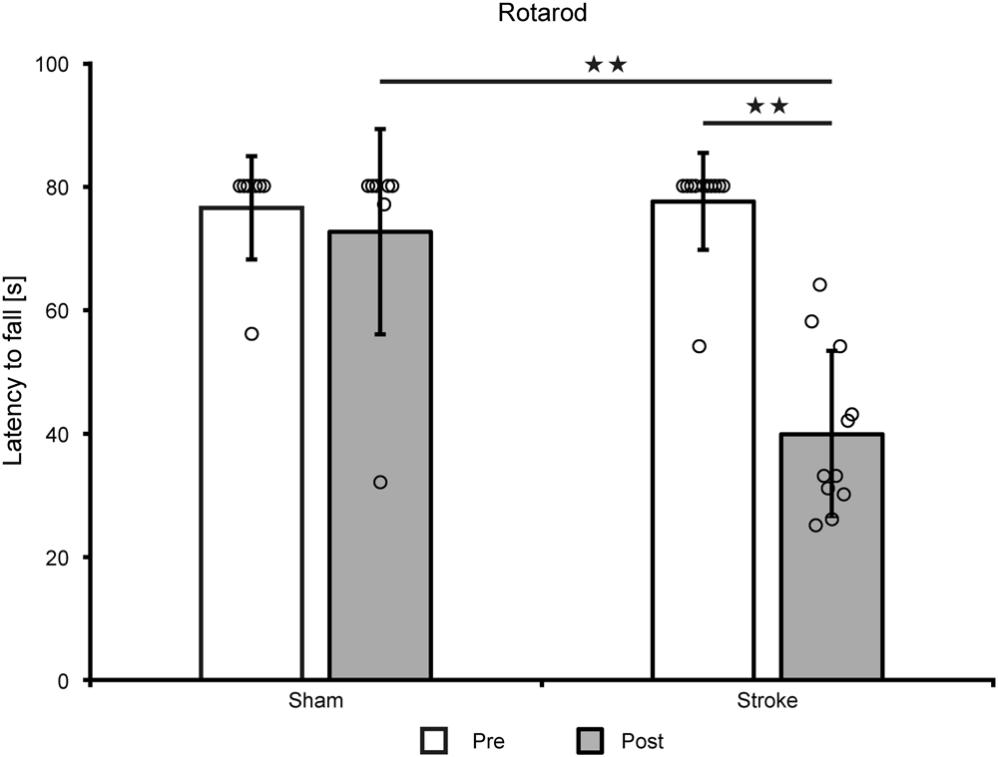
Rotarod motor test. Rotarod test at 1 week after dMCAO shows a highly significant motor performance reduction of the ischemic animals compared to the pre-stroke value (Z = −2.936; p = 0.003) and compared to the sham group (Z = −2.884; p = 0.004) while there was no difference between both groups before stroke induction (Z = −0.249; p = 0.804). All individual volume data of all animals at all time points are compiled in the Supplement Table 3. Error bars are presented as SD.

Balance on the rotarod was assessed before and one week after stroke induction (Fig. 6). A 2-tailed Wilcoxon Mann Whitney test showed that both, stroke group and sham group, elicit a statistically different performance at one week after stroke (Z = −2.884; p = 0.004). A 2-tailed Wilcoxon signed rank test was carried out to test the difference in performance within the same group, before and after stroke induction. Only the stroke group showed a highly significant difference when both time points were compared (Z = −2.936; p = 0.003), indicating the functional deficit after stroke.

**Figure 7:**
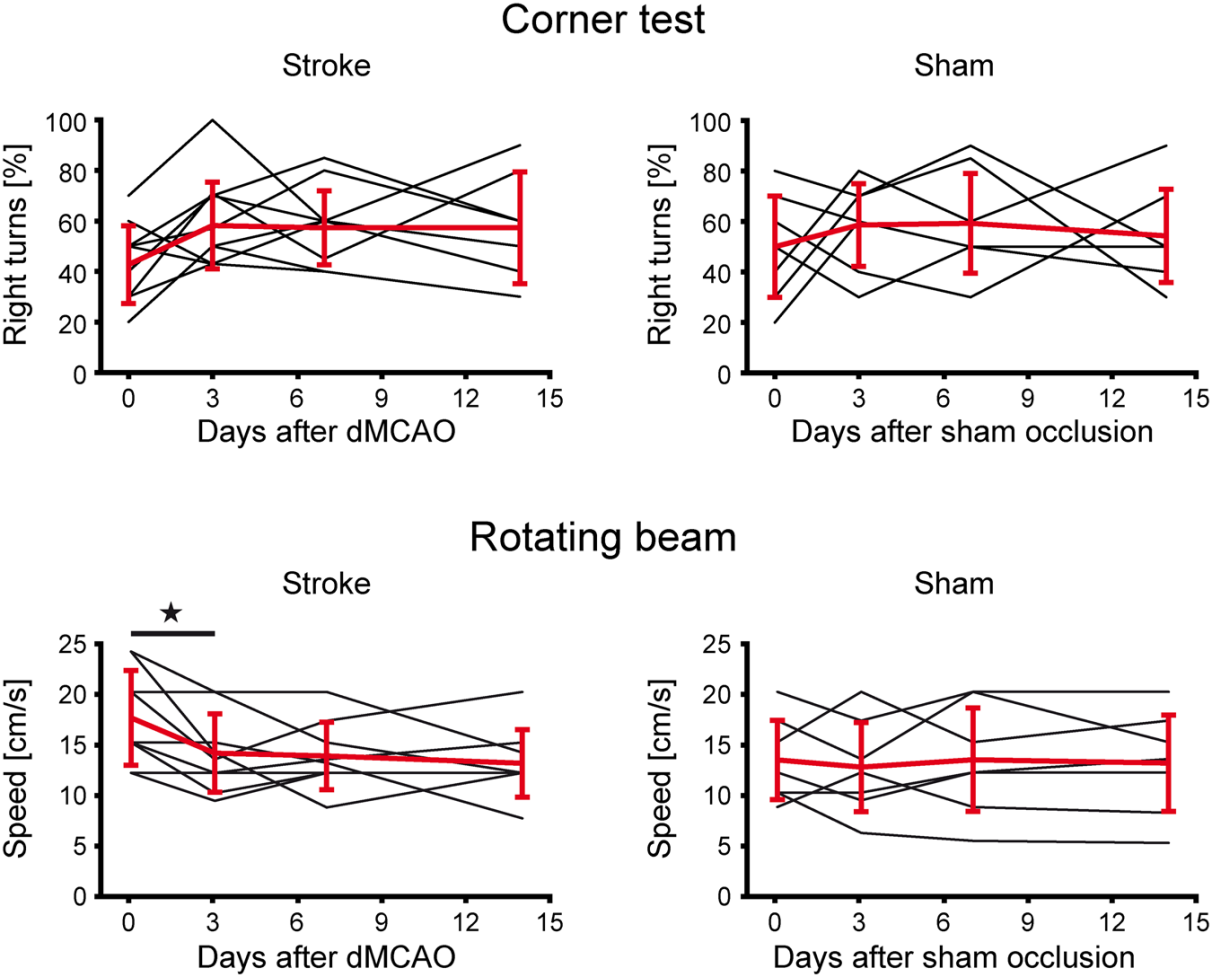
Corner and rotating beam test. The corner test (top row) a dn the rotating beam test (bottom row) were performed before stroke and at 3,7, and 14 days following distal MCA occlusion (left) or sham surgery (right). Corner test: The scatter in individual behavior was rather large. Already before stroke induction, no balanced situation between left- and right-turns was noted. Moreover, no difference was noted between ischemic and sham group. Rotating beam test: The speed of traversal of the rotating beam varied substantially among the animals before stroke. The sham group showed no clear change after sham occlusion. In the stroke group, while being heterogeneous, five animals showed an improvement after primary functional decline while three mice showed no improvement; one animal had no change in performance during the whole observation period. All individual volume data of all animals at all time points are compiled in the Supplement Table 4 (Corner test) and Supplement Table 5 (Rotating beam), respectively. In the rotating beam test, two mice in the stroke group did not cooperate during the pre-stroke phase so that no data are available on these two animals. Error bars are presented as SD.

The corner test (Fig. 7 top) showed rather high inter-individual spread of right- and left-turns for both groups (Suppl. Table 4). Intra-individual fluctuations over time made interpretations, even within the sham group, impossible and proved the test insensitive to changes induced by the cortical lesion. Group-averaged values showed no statistically relevant changes over time. This test, commonly successful with C57Bl/6 mice after stroke, reflects the reduced sensory dependence of the nude mice on their whiskers which are dramatically shorter compared to furry animals.

On the rotating beam, two animals were excluded from the stroke group due to a complete lack of performance cooperation during the pre-stroke period (i.e. they did not cross the beam at all). The other nine animals in the stroke group showed a rather heterogeneous behavior after stroke over time (Fig. 7 bottom; Suppl. Table 5). After a primary performance decrease after stroke induction, five animals exhibited a secondary improvement, one mouse showed no changes at all, and in the other three animals a continuous further decrease was noted. A statistically significant performance decrease was only noted for the first time point at 3 days. No change was observed for the sham occluded animals.

### Histological/immunohistochemical analysis

**Figure 8:**
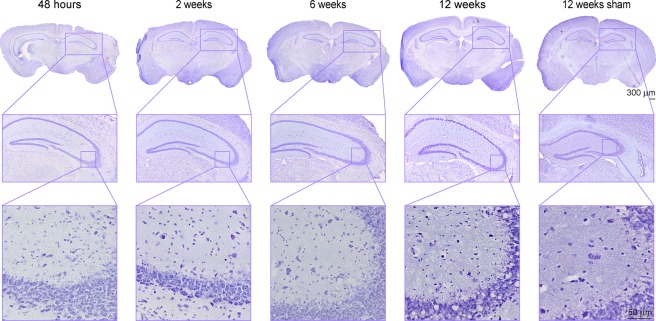
Nissl staining of cortical sections at the level of the hippocampus. Nissl staining of ischemic mice is presented of the whole brain section (4×) for different time points over the 12 weeks observation period, together with a representative sham animal at 12 weeks after the sham surgery. For better visualization the area of the hippocampus is enlarged at 20× magnification. A further zoom of the hippocampus region (60× magnification) was used for detection of pathological changes. A thinning of the cell density in the hippocampus becomes visible with time, followed by appearance of vacuolation at week 12. In comparison, the density of cell nuclei in the sham mouse shows no difference between both hemispheres and is free of vacuoles at 12 weeks after sham occlusion of the MCA.

Six additional mice with dMCAO were used for histological analysis with two animals for each time point (48 h; week 2; week 6) together with the histological data of all 18 animals at week 12. As the hippocampus apparently plays a major role in response to the primary cortical ischemia, we have analyzed tissue changes at the hippocampal level in more detail. Figure 8 presents the Nissl staining of ischemic mice at different times over the 12 weeks observation period, together with a representative sham animal at 12 weeks after the sham surgery. At higher magnification (20×), a slight thinning of the cell density in the hippocampus is visible, accompanied by appearance of vacuolation at week 12, best seen on 60× inserts. In comparison, the density of cell nuclei in the sham occluded mice shows no difference between both hemispheres and is free of vacuoles at week 12.

**Figure 9:**
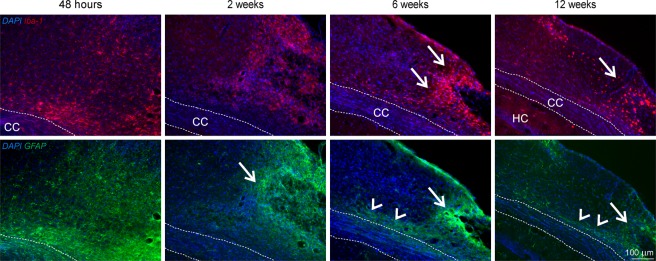
Immunohistochemical staining for inflammation and reactive gliosis. The upper row presents Iba1 positive activated microglia and infiltrated macrophages in the cortical lesion periphery. Distribution of Iba1-positive microglia and macrophages is diffuse in the cortex during early time points but becomes gradually more focused next to the coagulation site starting at week 6 and persisting until week 12 (arrows). In the lower row, GFAP staining shows reactive astrocytes forming a glial scar around the lesion (arrows) and spreading in the cortex along the corpus callosum (arrow heads). Abbreviations: CC: corpus callosum; HC: hippocampus.

Adjacent to the cortical ischemia, accumulation of activated microglia and infiltrated macrophages, represented by Iba1 IHC staining (Fig. 9, upper row), is already noted at 48 h. This inflammatory reaction further increases at week 2 and persists at this high level during the 12 weeks observation period. The distribution of Iba1-positive microglia and macrophages is diffuse in the cortex during early time points but becomes gradually more focused next to the coagulation site starting at week 6 and persisting until week 12. Reactive gliosis presented by GFAP staining is already visible at 48 h post occlusion, where astrocytes can be recognized spreading over the ischemic tissue (Fig. 9, lower row) and are later gradually recruited towards the lesion site and above the corpus callosum.

## Discussion

We have characterized the long-term lesion development and morphological changes after distal MCA occlusion in the chronic phase of stroke in immune-deficient nude mice. To our knowledge, this is the first study unraveling the pathophysiological alterations of this dMCAO mouse model in a time window that is relevant for neurorepair or regeneration studies such as after stem cell treatment. Most prominent findings of this investigation are the cortical tissue loss reaching 15% of the pre-stroke cortex volume and the hippocampal volume increase by 10%, resulting in a lateral shift of the ipsilateral hippocampus, moving together with the corpus callosum into the cortical tissue void and onto the brain surface.

The morphological changes of cortical tissue loss become apparent already at 2 weeks after dMCAO, visualized as cortical thinning on the MR images. This effect progresses over time with maximal tissue loss of approximately 15%, already reached at week 6. This tissue void makes room for subcortical tissue. In this course, the massively swelling hippocampus together with the corpus callosum is shifted to the brain surface, showing an almost radial direction of the corpus callosum towards the brain surface. The hippocampus volume, calculated from the MRI images before stroke induction, resulted in 12 mm3 for one hemisphere, which is in good agreement with earlier reports on mouse brain morphometry by Baeda *et al*.^11^, describing approximately 26–28 mm3 for the whole mouse brain, with an inter-individual variability of only 5% hippocampus volume. Thus, our findings of 10% hemispheric hippocampal volume increase is not only statistically significant but also clearly beyond the inter-individual variations. Earlier investigations had covered only the acute or subacute phase of stroke development after dMCAO. In some reports, the lesion is characterized by triphenyltetrazolium chloride (TTC) staining or histology at 24 to 72 h^2,6,8–10^, in one further report, lesion volume was determined by TTC at various times during the first week^3^. Only in two reports from the same lab, the lesion development was described by MRI during the first three weeks^4,7^. All these studies have in common that they are restricted to maximally three weeks when the most dramatic structural changes of hippocampal swelling and corpus callosum pushing are only starting to develop and the changes in morphology can be only poorly recognized. Furthermore, most studies had only focused on the ischemic alterations of existing tissue on TTC staining without analyzing cortical tissue loss. In two reports from one lab “loss of tissue” was mentioned but without quantitatively analyzing the loss^2,6^. Two further studies reported on lesion volumes at 3 and 8 weeks, respectively^5,12^. Freret *et al*. found^5^ a lesion of 10–15 mm3 at three weeks, closely similar to our values observed at 6 weeks while Shen *et al*.^12^ described an atrophic volume after 60 days of 13 ± 6%, in full concordance with our own value of 15 ± 5%. However, both these studies focused on other aspects like behavioral tests or vessel density changes, but neither analyzed the structural changes and tissue rearrangements underlying the reported lesion volumes. Thus, our report is the first to describe the cortical tissue loss and the consequential rather complex tissue rearrangement massively involving displacement of the hippocampus and the corpus callosum. We also demonstrate that careful handling of the procedures during sham occlusion reliably prevents any of these serious morphological and tissue defects. Hence, the above described structural rearrangements are a consequence of the ischemic change and not of the surgical intervention or the forceps damage.

It is noteworthy that the massive structural alterations found here are novel and have not been described before in the dMCAO model. These pronounced changes are particularly surprising as other models such as the transient filament occlusion model, known to produce large ischemic territories, do not produce hippocampal movement and corpus callosum squeezing across the cortical region to the surface. The structural rearrangements in the brain induced by the dMCAO model must be emphasized as this stroke model is widely assumed to produce only small, focused cortical damage. Thus, the elicited damage visible at two weeks after stroke induction on T2-weighted MRI gives the wrong impression of a focal lesion with only cortical involvement. Instead, attention must also be paid to later time points. Applications of this dMCAO model for stem cell mediated regeneration studies will have to carefully consider the grafting location which is usually chosen outside but adjacent to the ischemic lesion, in order to avoid jeopardizing the graft vitality by delayed further expansion of the tissue deterioration^1^ or even structural rearrangements as described here. Furthermore, future studies will have to assess whether the structural rearrangements of this stroke model may result in altered functional network connectivities and even structural network changes.

Quite a few earlier investigations have reported on behavioral deficits of the dMCAO mouse model^5,6,8–10,12^. Our behavioral tests aimed to assess the functional deficit in the acute phase as a functional confirmation of successful stroke induction. Our results showed variable sensitivity to the ischemic impact. The corner test was found useless, presumably due to the lack of substantial vibrissae density and length in nude mice. Moreover, this test was reported to have highly variable results also in some other mouse strains^10^, while Shen and colleagues^12^ found it useful in transgenic mice and corresponding WT littermates. Testing FVB and Swiss mice, these former authors also did not find significant differences on either the rotarod or the beam walk. The rotarod test in our hands on our nude mice demonstrated highly significant difference at one week after stroke onset between sham occluded mice and those submitted to dMCAO. Our rotating beam test, being closely similar to the beam walk test used by Rosell and colleagues^10^, resulted in rather heterogeneous inter-individual behavior patterns over time and, in consequence, led to significant performance reductions in dMCAO mice only during the first three days. In summary, the rotarod test appears the most sensitive and homogenous test to be used for assessment of functional deficit after distal MCA occlusion in nude mice.

Our characterization of the cortical infarct by pannecrosis and vacuolation is in agreement with earlier descriptions by Kuraoka *et al*. at day 4 and 8 after dMCAO^3^. We have shown that the inflammatory response to the cortical stroke induction starts with the recruitment of Iba1 positive cells in and around the ischemic territory already at 48 h, increasing over time and persisting during the whole 12 weeks of observation. This is in agreement with Kuraoka’s finding of Iba1 positive cell increase from 24 h to four days^3^. Finally, the observed increase of the glial scar with GFAP-positive reactive astrocytes around the ischemic lesion has also been reported by Caballero-Garrido and colleagues^4^ and by Kuraoka *et al*.^3^ for the early time window of one week after distal MCA occlusion, and is similarly found in the filament occlusion model already at 2 weeks post occlusion^13^.

## Conclusions

Early detection of the affected tissue by MRI in the acute time severely overestimates the final cortical lesion extent, showing more than twice the (edematous) volume than the atrophic volume, determined at week 6 to 12. We have shown that the distal MCA occlusion model in the immune-deficient nude mouse leads to delayed cortical thinning resulting in final cortical tissue loss of 15% in the chronic phase of stroke. In parallel, a substantial swelling and consequent displacement of the hippocampus was observed, pushing the corpus callosum into the void of cortical tissue. Knowledge of the chronic morphological changes and rearrangements, not known in other stroke models, is highly important for experimental protocols of long-term studies where neurorepair or regeneration aspects are to be studied. Only thus, a safe placement of stem cell grafts can be assured, thereby avoiding delayed lesion expansion into the graft location, jeopardizing the stem cell vitality.

## Materials and Methods

### Animal handling

A total of 36 homozygous male NMRI-Fox1^nu^/Fox-1^nu^ mice with Tyr^c^ albino background were used for all experiments. The autosomal recessive mutation in the Foxn1 gene (chromosome 11) causes thymic aplasia, which results in immunodeficiency. As a consequence, there is a lack of T cells, but B cells remain, which makes this strain ideal to host allograft implants. Mice were purchased from Janvier (Le Genest-Saint-Isle, France) and housed under a 12:12 hours light:darkness cycle, resting in quarantine for one week after arrival before entering experiments. Mice had access to food and water *ad libitum*, and 3–4 individuals shared the same cage for the purpose of social housing. At the first week of the longitudinal experiment, mice were 8 weeks old and weighted 30 ± 2 grams.

All animal experiments were conducted in accordance with the German Animal Welfare Act guidelines and approved by the local veterinarian authorities from LANUV (Landesamt für Natur, Umwelt und Verbraucherschutz Nordrhein Westfalen). The animal permission was approved under the number 84–02.04.2014.A370.

### Experimental protocol

**Figure 10:**
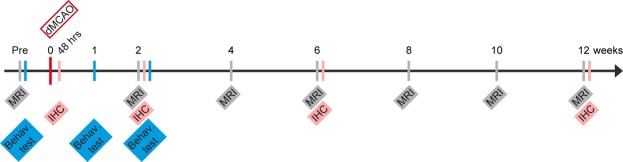
Schematic of the experimental protocol. MR imaging and behavior tests were performed before stroke induction and at various time points during the 12 weeks after distal occlusion of the middle cerebral artery (dMCAO). Behavior tests were limited to the first two weeks of ischemia, the period sensitive to primary ischemic damage. Histology was performed at 48 h, week (w)2, 6, and 12 after dMCAO.

The schematic time profile of the experimental protocol is depicted in Fig. 10. MR imaging and behavior tests on 18 mice were performed before stroke induction and at various time points during 12 weeks after distal occlusion of the middle cerebral artery. Behavior tests were limited to the sensitive period at the first two weeks of ischemia. Histology was performed at the end of the 12 weeks observation period and six additional mice were included to obtain material for histology also at 48 h, week 2, and week 6 after dMCAO with two mice for each time point. For the control group, 12 sham animals were included and received the identical protocol as the stroke animals except for the stroke induction.

### Stroke induction

The animals were anesthetized in a knock-out box with an isoflurane flow rate of 4% in 70%:30% N2O:O2 atmosphere. Subsequently, the animals were placed on a warm plate to keep body temperature stable during surgery, while the mouse head was placed laterally in the anesthesia mask and isoflurane concentration was lowered to 2%. Breathing rate was monitored and kept above 80 bpm at all times. Eye ointment with Bepanthen (5% Dexpanthenol; Bayer Vital, Leverkusen, Germany) was applied to prevent the eyes from drying. Rimadyl (50 mg/ml Carprofen, Zoetis, Berlin, Germany), diluted 1:100 in saline, was injected to induce analgesia, in a final dose of 4 mg/kg body weight. After checking for pain reflexes, the skin on the right side of the head was sterilized with ethanol and a 1 cm incision was made between the ear and the eye. The skin was pushed to the side and the temporal muscle exposed. The electrocoagulation forceps were used at 12 Watt (W) to detach the muscle from the skull in its apical and dorsal parts, with the intention to make a flap to expose the bone. The skull was cleared from tissue and the distal end of the middle cerebral artery was identified through the translucent bone in the rostral part of the temporal area, rostral to the retro-orbital sinus. Some drops of saline were applied on the skull and the bone was drilled thin above the intended coagulation site. When the bone layer became transparent, the remaining bone sheet was removed manually with the help of a thin forceps. Once the artery was exposed, the electrocoagulation forceps were set to 7 W (bipolar) and the artery was coagulated from both sides without grasping it to avoid a rupture and subsequent hemorrhage. Thirty seconds after coagulation, the artery was cut to confirm that reperfusion did not occur. The temporal muscle was flapped back into position, thereby covering the bone hole. The skin was sutured with silk thread (RESORBA Medical, Nürnburg, Germany). Analgesia was maintained with Tramal (100 mg/ml Tramadol hydrochloride, Grünenthal, Stolberg, Germany) in drinking water (1 mg/ml) for the following 3 days.

For the sham surgery, the same steps were followed but the forceps tips were kept more separated from each other to avoid coagulation of the MCA.

In both groups, no unintended mortality was observed, i.e. all animals reached the intended endpoint after 12 weeks.

### Histology and Immunohistochemistry

Mice were perfused transcardially under 4% isoflurane anesthesia in 70%/30% N2:O2 atmosphere with 20 ml PBS followed by 20 ml 4% paraformaldehyde (PFA) solution. The brains were carefully removed with a surgical spatula and incubated overnight in 4% PFA solution at 4 °C. On the following day, the PFA was replaced by 30% sucrose solution and incubated for 2 days at 4 °C. On the third day, brains were shock-frozen in −40 °C cold 2-methylbutane and rapidly transferred into −80 °C storage. Sections of 14 or 30 μm thickness were cut in the coronal plane with the cryostat (Leica Biosystems, Wetzlar, Germany) and stored at −20 °C.

For the detection of Iba1- and GFAP-positive cells, immunohistochemical (IHC) staining was conducted. Tissue sections were washed 3× for 5 min in PBS, and antigen retrieval was conducted by incubation in −20° cold acetone for 20 min at 4 °C. Subsequently, tissue sections were washed again 3× for 5 min in PBS and brain sections were rimmed with a PAP Pen to limit the volume of the applied solutions. To avoid non-specific binding of antibodies, sections were incubated in blocking solution containing PBS, 0.25% Triton X-100, and 5% normal donkey serum for one hour at room temperature. The primary antibodies were diluted in the blocking solution and incubated overnight at 4 °C in a wet chamber for at least 18 h. The following primary antibodies were used: mouse anti GFAP (1:200; Sigma) and rabbit anti Iba1 (1:500; Wako). On the following day, tissue sections were washed 3× for 3 min in PBS. The secondary antibodies were diluted together with the nuclear stain Hoechst 33342 in blocking solution and incubated in a wet chamber at room temperature for 2 h. Cy5 donkey anti mouse (1:200; Jackson ImmunoResearch) and Cy3 donkey anti rabbit (1:200; Jackson ImmunoResearch) were used for GFAP and Iba1, respectively. Subsequently tissue sections were washed again 3× for 5 min with PBS followed by distilled water, PAP Pen was removed with ethanol, and sections were mounted with Cytoseal XYL (Thermo Fisher Scientific, Waltham (MA), USA).

For Nissl staining, tissue sections were rehydrated by incubation in a descending alcohol series in aqueous isopropanol dilutions ranging from 96 to 60% and finally pure distilled water. The Nissl staining was conducted with a ready-to-use Nissl staining Kit (MORPHISTO, Frankfurt am Main, Germany). For this reason, the tissue sections were placed in cresyl violet (1:5 diluted with distilled water) for 40 sec and washed for 1 min in distilled water. Finally, the tissue was dehydrated and differentiated in ascending alcohol series and finally with Roti-Histol (Carl Roth, Karlsruhe, Germany). Tissue sections were covered with Cytoseal XYL and left to dry overnight.

### Behavioral tests

Within the present investigation of elucidating the structural reorganizations after distal middle cerebral artery occlusion in nude mice, the performance of the behavior tests had the subordinate function to elucidate which tests are sensitive to this stroke model in nude mice. The most sensitive test will be considered for future therapeutic studies using stem cell mediated regeneration after stroke. Therefore, only a reduced number of animals was included into the behavior tests, with 11 of 18 in the stroke group and 7 of 12 in the sham group.

#### Rotarod test

The set-up consists of a rotating cylinder of 3 cm diameter with four compartments for parallel testing of several mice. The paradigm for the motor deficit assessment after stroke included a gradual acceleration to 30 rounds per minute (rpm) during the first 50 s, followed by constant speed for another 30 s. The test was terminated after 80 s. Mice were evaluated for latency to fall, reported in seconds. The rotarod test was performed at two time points of the longitudinal experiment: before dMCAO (control) and one week after stroke induction, as described by Balkaya and colleagues^14^.

#### Rotating beam test

The apparatus consists of a rotating fiberglass beam of 120 cm length and 13 mm diameter, with distances marked every 10 cm. The beam is rotated by a motor and is located 60 cm above a table covered with bubble cushions. On the arrival platform at the end of the beam, a food pellet was placed to motivate the mice to walk forward and not turn back. Mice were pre-trained at days 8, 6 and 4 before stroke induction, and the last record was set as baseline speed for comparison after stroke. The beam was set to rotate at 6 rpm at constant speed, the animal was placed on the departing end of the beam in motion and left to walk to the arrival end. The total time until the animal was safely located on the arrival platform was recorded. In the case of a fall, a penalty of 60 s for the drop was added to the time of the second, ssuccessful trial. Rotating beam tests after stroke induction were obtained at days 3, 7 and 14 for both, the stroke and the sham occluded group.

#### Corner test

The set-up consists of four connected varnished wooden walls placed in the shape of a diamond with 30 and 150 angles, respectively. Each wall measures 30 × 20 × 1 cm. At one of the 30 degree corners, a small opening is left to motivate the mice to explore further and thereby go deeper into the corner. The animal was carefully placed in the middle of the open space inside of the diamond-shaped set-up and left to explore the corners freely. At some point after entering the corner, the animal would rear and turn to the left or right side. There was no time limit to record the moves. The first 10 successful turns after approaching the narrow corners were recorded and noted as “right turn” or “left turn”. Turns that were not part of a rear movement were ignored. A baseline measurement was done before induction of stroke to compare intra-animal tendency to turn to one or other side. The results of the corner test were expressed as % of the total number of tests. Measurements were taken for the stroke and sham occluded group at days 3, 7 and 14 after stroke induction.

### MRI data acquisition

MRI measurements were performed with a 9.4 Tesla animal scanner (Bruker Biospin, Ettlingen, Germany) with 20 cm horizontal bore diameter, equipped with actively shielded gradient coils. RF transmitting and signal receiving was performed with a helium-cooled mouse 1 H quadrature cryogenic surface coil (CryoProbe, Bruker Biospin). Paravision 6.1 (Bruker Biospin) was used to control the MRI acquisition protocols. The animal was placed in an animal holder equipped with a mask supplying breathing gases. The mouse head was fixed with teeth and ear bars. The animal’s body temperature was measured via a fiber optic rectal probe (1025 T System, SA Instruments, Stony Brook, NY, USA) and kept constant at 37 ± 1.0 °C by an adjustable water circulating system (medres, Cologne, Germany). Additionally, breathing rate was continuously monitored (1025 T System) and recorded (DASYlab Software, Measurement Computing, Norton, USA).

A 3-plane pilot reference scan using FLASH was acquired to confirm correct positioning of the mouse head. Next, a high-resolution anatomical T2-weighted TurboRARE sequence was acquired with a RARE factor of 8 and two averages. The field-of-view (FOV) was set to 17.5 × 17.5 mm^2^ and 48 slices of 0.3 mm thickness without gap were placed to cover the whole mouse brain. The matrix dimension was set to 256 × 256, repetition time (TR) to 5,500 ms and echo spacing to 10.8 ms, resulting in an effective echo time (TE) of 32.5 ms. In-plane resolution was (68 μm)^2^ and a bandwidth of 39062 Hz. A multi-slice multi-echo (MSME) sequence was recorded for T2 evaluation and visualization of hyperintense signal in the brain. MSME acquisition parameters were as following: TE = 11 ms, TR = 3,000 ms, number of echoes = 16, scan time of 6 min 24 sec, number of averages was 1, matrix size of 128 × 128, FOV of 18 × 18 mm, in-plane spatial resolution of (140 μm)^2^, bandwidth of 50.000 Hz, slice thickness of 0.5 mm, number of slices was 11, coronal orientation with a slice gap of 0.5 mm.

### MRI data processing

#### T2 incidence maps

In order to assess the stability and reproducibility of the induced stroke lesion, an incidence map of the lesion extent across the group of all 18 animals was determined at 48 hours after stroke. First, T2 maps of each animal were calculated from the MSME scan using custom written program in IDL software (Exelis Visual Information Solutions, Boulder, CO, USA). For each subject, the first echo of the MSME scan was linearly co-registered with the corresponding anatomical TurboRARE image using FSL flirt (FMRIB Software Library; http://www.fmrib.ox.ac.uk/fsl)^15,16^. Then, the anatomical data were co-registered to an in-house created template of 8 weeks old nude mice (n = 21)^1^. The two transformation matrices were combined and applied to the T2 maps, resulting in linearly transformed T2 maps. The T2 value of the healthy contralateral cortex was determined and a T2 threshold at 15% or more above the normal T2 value of cortex was set for reliable discrimination of the ischemic lesion. Further, a mask was created with ImageJ software (Version 1.46; National Institutes of Health, Bethesda, USA; http://rsbweb.nih.gov/ij) by thresholding to exclude the ventricles of each individual subject. From both, the co-registered T2 maps and the mean T2 threshold of ischemic tissue, stroke incidence maps were generated using in-house ImageJ macros.

#### Determination of lesion volume

In the here described mouse model of distal MCA occlusion, the effective lesion encompasses i) the cortical tissue denoted by hyper- and hypo-intense areas and ii) the atrophic volume of lost tissue due to resorption of necrotic tissue. A further prominent feature of the structural changes was the hippocampal volume increase during the 12 weeks observation period. Due to the continuously increasing, massive morphological changes of the brain during the 12 weeks observation period after stroke induction, the following complex procedures became necessary for the quantification of the cortical tissue. Using ANTs (http://stava.github.io/ANTs) (Avants *et al*., 2011), first a linear coregistration of the anatomical data (TurboRARE) onto the template was performed. The linearly transformed TurboRARE data was segmented into three sets: manual segmentation of the hippocampus, remaining brain (template mask) and background. A coarse nonlinear point set expectation (PSE) registration of the segmented TurboRARE images onto the segmented template results in a PSE warp field which was applied to the TurboRARE images. A subsequent nonlinear coregistration of the transformed anatomical data (TurboRARE) onto the template was performed, followed by a nonlinear coregistration of the template onto publicly available mouse brain atlas of the neocortex (Ullmann *et al*., 2013). The transformation matrices and warp fields were combined and the inverse transformation was executed, mapping the cortex labels of the mouse brain atlas onto the original, unchanged anatomical data. The voxels of the contralateral, normal cortex and of the thinned and distorted ipsilateral cortex were summed up to determine the cortex volume of both hemispheres using fslstats (FSL). Lost cortical tissue volume was calculated as the difference between the ipsilateral cortex volume before stroke minus the cortex volume at the time point of interest after stroke induction.

From the manual hippocampus segmentation and the coregistration, the ipsilateral hippocampal volume was calculated at all time points, before stroke and at 2, 6, and 12 weeks after stroke induction.

Hyper- and hypo-intense areas were present in particular during the early stroke periods and became increasingly patchy with time. Therefore, these regions were manually delineated in all slices for each time point from 2 to 12 weeks and the volume of pathological image intensity was calculated.

### Statistics

For behavioral tests and cortical and hippocampal volume determination, ANOVA for repeated measurements was performed (SPSS Statistics version 22, IBM Corp., USA), assuming sphericity in the distribution. After checking for statistically significant differences within repetitive measures in the same group, a post hoc Bonferroni test was chosen to check differences among the groups at the same time point. For the rotarod data a nonparametric test (two-tailed Wilcoxon Mann Whitney) was used to test for success of remaining on the rotarod for the defined period of 80 s. A *p*-value ≤ 0.05 was considered to be significant (*), and *p* ≤ 0.005 was considered to be highly significant (**). All error bars are expressed as standard deviation (SD) unless stated otherwise.

## Supplementary information

Supplementary Data

## Data Availability

Raw data will be made available via a data repository upon acceptance of manuscript.
