# Supplementary material for: Cortical tissue loss and major structural reorganization as result of distal middle cerebral artery occlusion in the chronic phase of nude mice
Source: Sci Rep. 2019 May 2;9:6823. doi: 10.1038/s41598-019-43341-0 (PMC6497904; doi:10.1038/s41598-019-43341-0)
Supplement: Supplementary file 1 — Supplementary Data [file 41598_2019_43341_MOESM1_ESM.pdf]

Supplementary Materials

Cortical tissue loss and major structural reorganization as result of distal middle cerebral artery occlusion in the chronic phase of nude mice

Anuka Minassian[1], Marina Dobrivojevic Radmilovic[1], Stefanie Vogel[1], Michael Diedenhofen[1], Melanie Nelles[1], Maren Stoeber[1], Dirk Wiedermann[1], Mathias Hoehn[1,2,*]

[1]In-vivo-NMR Laboratory, Max Planck Institute for Metabolism Research, Cologne, Germany

[2]Department Radiology, Leiden University Medical Center, Leiden, Netherlands

Supplementary Table 1

Cortex volume (mm^3)

Stroke group

	Pre-stroke			48 hours			2 weeks			6 weeks			12 weeks		
Animal#	LC	RC healthy	RC Lesion	LC	RC healthy	RC Lesion	LC	RC healthy	RC Lesion	LC	RC healthy	RC Lesion	LC	RC healthy	RC Lesion
Stroke#1	61	60	0	61	38	24	61	48	6	59	48.6	0.8	60	52	0.5
Stroke#2	68	68	0	67	43	24	68	54	8	67	56.0	1.4	63	55	0.5
Stroke#3	61	61	0	60	44	16	61	49	6	60	51.1	1.2	65	56	0.8
Stroke#4	67	67	0	67	44	23	67	50	10	65	51.6	1.8	63	54	0.6
Stroke#5	64	65	0	62	51	12	64	49	9	63	54.6	1.1	69	57	0.3
Stroke#6	67	67	0	65	57	8	67	57	5	65	55.5	1.1	63	54	0.36
Stroke#7	70	70	0	64	36	28	64	52	6	63	52.4	0.9	66	60	0.1
Stroke#8	70	70	0	68	35	33	69	49	9	67	53.8	0.9	64	55	0.2
Stroke#9	65	65	0	67	44	23	63	49	8	62	51.1	1.0	69	64	0.2
Stroke#10	66	66	0	69	56	12	69	62	3	68	62.5	0.6	69	58	1.6
Stroke#11	67	67	0	63	45	20	70	63	4	65	55.7	1.4	68	62	0.3
Stroke#12	70	70	0	71	36	35	72	57	2	69	63.0	0.4	71	63	0.4
Stroke#13	72	72	0	69	29	40	71	63	8	70	56.7	3.5	68	61	0.3
Stroke#14	70	70	0	71	34	36	74	66	3	69	62.9	1.0	72	65	0.4
Stroke#15	75	74	0	59	27	32	75	66	4	72	64.9	1.5	70	62	0.2
Stroke#16	76	76	0	66	21	45	75	68	5	71	62.2	1.7	67	61	0.1
Stroke#17	75	75	0	62	30	33	72	62	4	71	62.6	1.6	65	59	0.4
Stroke#18	70	71	0	70	54	16	72	62	4	65	56.8	1.3	69	57	0.7
Average	69	69	0	66	40	26	69	57	6	66	57	1.3	67	59	0.4
SD	4.26	4.26	0	3.63	10.04	10.14	4.42	6.89	2.15	3.78	4.88	0.65	3.20	3.83	0.33

Abbreviations: LC = left cortex; RC = right cortex; SD = standard deviation

Supplementary Table 2

Hippocampus volume (mm^3)				
		**Stroke group**		
Animal #	Pre-stroke	2 weeks after stroke	6 weeks after stroke	12 weeks after stroke
Stroke#1	12.11	12.39	12.36	12.44
Stroke#2	12.62	13.27	13.53	14.15
Stroke#3	12.38	12.14	12.64	12.71
Stroke#4	13.17	12.73	12.90	13.12
Stroke#5	13.38	13.76	13.76	13.48
Stroke#6	13.24	12.84	13.20	13.13
Stroke#7	12.28	13.60	13.76	14.19
Stroke#8	10.63	11.24	10.27	10.76
Stroke#9	12.06	12.26	12.74	12.93
Stroke#10	10.69	11.31	11.41	12.05
Stroke#11	11.55	12.34	13.13	14.09
Stroke#12	11.40	11.37	11.95	12.32
Stroke#13	11.83	12.52	12.59	12.55
Stroke#14	11.46	11.77	11.50	12.29
Stroke#15	12.15	13.20	14.51	12.32
Stroke#16	11.45	11.95	12.18	12.35
Stroke#17	12.01	11.77	11.60	11.45
Stroke#18	10.68	11.26	11.64	11.66
Average	11.95	12.32	12.54	12.67
Standard deviation	0.81	0.78	1.01	0.91

Supplementary Table 3

Latency to fall on rotarod (s)					
**Stroke**			**Sham**		
Animal Number	Before stroke	After stroke	Animal Number	Before stroke	After stroke
Stroke#1	80	58	Sham#1	80	80
Stroke#2	80	25	Sham#2	80	80
Stroke#3	80	64	Sham#3	80	80
Stroke#4	80	33	Sham#4	56	32
Stroke#5	54	31	Sham#5	80	77
Stroke#6	80	26	Sham#6	80	80
Stroke#7	80	33	Sham#7	80	80
Stroke#8	80	54			
Stroke#9	80	30			
Stroke#10	80	42			
Stroke#11	80	43			
Average	77.6	39.9	Average	76.5	72.7
Standard deviation	7.8	13.5	Standard deviation	8.4	16.7

Supplementary Table 4

Corner test (Right turns in % of total tests)				
		**Stroke group**		
Animal #	Pre-stroke	3 days after stroke	7 days after stroke	14 days after stroke
Stroke#1	60	40	40	30
Stroke#2	30	40	60	90
Stroke#3	50	40	40	30
Stroke#4	20	50	60	40
Stroke#5	50	60	80	60
Stroke#6	70	100	60	90
Stroke#7	30	70	40	80
Stroke#8	40	70	60	50
Stroke#9	50	70	80	60
Stroke#10	50	40	60	70
Stroke#11	20	50	40	30
Average	42.73	57.36	56.36	57.27
Standard deviation	15.43	18.20	14.32	22.19
		**Sham group**		
Animal #	Pre-sham occlusion	3 days after sham occlusion	7 days after sham occlusion	14 days after sham occlusion
Sham#1	50	30	50	40
Sham#2	40	80	60	90
Sham#3	60	40	30	70
Sham#4	20	60	50	50
Sham#5	80	70	90	50
Sham#6	30	70	80	30
Sham#7	70	60	50	50
Average	50.00	58.57	58.57	54.29
Standard deviation	20.00	16.41	18.84	18.41

Supplementary Table 5

Rotating beam test (Traversal speed cm/s)				
		**Stroke group**		
Animal #	Pre-stroke	3 days after stroke	7 days after stroke	14 days after stroke
Stroke#1	24	14	8.57	12
Stroke#2	20	20	15	12
Stroke#3	15	12	13.33	15
Stroke#4	12	12	12	12
Stroke#5	20	13.33	17.14	20
Stroke#6	24	20	20	14
Stroke#7	15	10	12	12
Stroke#8	15	15	13	7.5
Stroke#9	12	9.23	12	12
Stroke#10	N/D	N/D	N/D	N/D
Stroke#11	N/D	N/D	N/D	N/D
Average	17.44	13.95	13.67	12.94
Standard deviation	4.69	3.88	3.33	3.34
		**Sham group**		
Animal #	Pre-sham occlusion	3 days after sham occlusion	7 days after sham occlusion	14 days after sham occlusion
Sham#1	15	20	15	17.14
Sham#2	10	10	12	13.33
Sham#3	17.14	13.33	20	20
Sham#4	20	17.14	20	15
Sham#5	12	9.23	12	12
Sham#6	10	6	5.21	5
Sham#7	8.57	12	8.57	8
Average	13.24	12.53	13.26	12.93
Standard deviation	3.93	4.44	5.12	4.78

N/D: Data not determined as animals did not cooperate during pre-stroke tests.

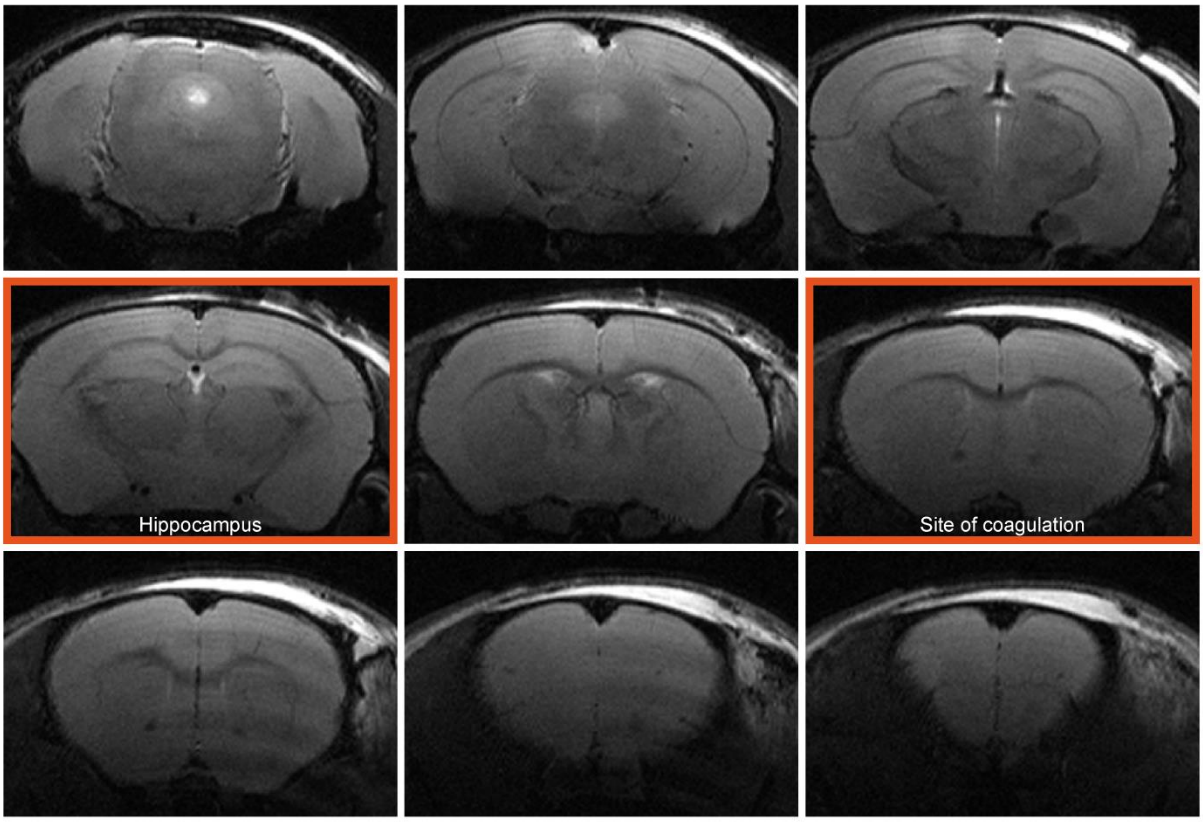

Supplementary Figure 1: Anatomic MR images of sham occluded mice at the acute time point.

A representative example of T2-weighted MRI of a mouse at 48 hours after sham surgery shows both hemispheres equivalent with no sign of damage. Only at the cortical surface at the level of the sham coagulation site, a minute hyperintense spot is marked indicating a weak reaction to the hot forceps touching the brain surface during sham surgery. The image planes at the site of coagulation in the ischemic animals and at the level where the hippocampal distortion is maximal in the ischemic animals are marked with a red frame. These two planes are used for further detailed analysis of any potential temporal changes (cf. Suppl. Fig. 2).

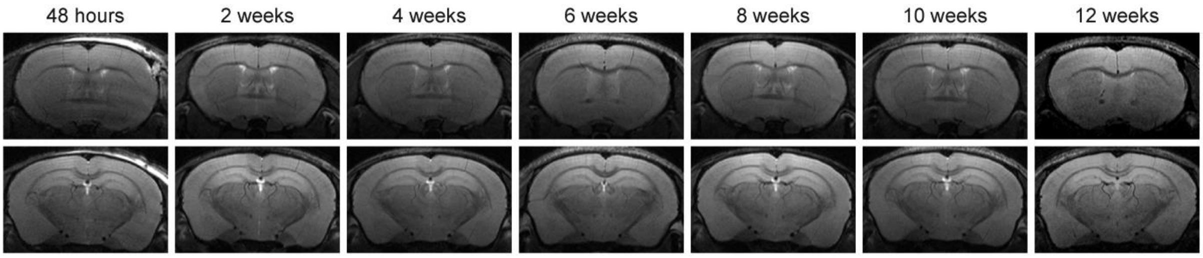

Supplementary Figure 2: Anatomic MR images of sham occluded mice during 12 weeks after sham surgery.

T2-weighted MR images of two selected coronal positions (corresponding to the sections depicted in Fig. 3 for the ischemic animals) through the brain of a representative mouse are shown at 48 h and for time points every two weeks following sham surgery. The slice positions were selected as described in Fig. 2 and correspond to the marked slices in Suppl. Fig. 1. The small hyperintense spot at 48 h quickly fades away. No hippocampal movement towards the brain surface is visible. In the upper row of images at the level of the sham dMCA coagulation, no cortical thinning and no bending of the corpus callosum towards the brain surface is detected (cf Fig. 3).
